# Pivotal role of DPYSL2A in KLF4-mediated monocytic differentiation of acute myeloid leukemia cells

**DOI:** 10.1038/s41598-020-76951-0

**Published:** 2020-11-20

**Authors:** Mina Noura, Ken Morita, Hiroki Kiyose, Hidemasa Matsuo, Yoko Nishinaka-Arai, Mineo Kurokawa, Yasuhiko Kamikubo, Souichi Adachi

**Affiliations:** 1 Department of Human Health Sciences, Graduate School of Medicine, Kyoto University, 53 Kawahara-cho, Syogoin, Sakyo-ku, Kyoto, 606-8507 Japan; 2 Department of Pediatric Oncology, Dana-Farber Cancer Institute, Harvard Medical School, Boston, MA 02215 USA; 3 Center for iPS Cell Research and Application (CiRA), Kyoto University, Kyoto, 606-8507 Japan; 4 Department of Hematology and Oncology, Graduate School of Medicine, The University of Tokyo, Bunkyo-ku, Tokyo, 113-8655 Japan; 5 Department of Pediatrics, Graduate School of Medicine, Kyoto University, Sakyo-ku, Kyoto, 606-8507 Japan

**Keywords:** Leukaemia, Acute myeloid leukaemia

## Abstract

Although the biological importance of Krüppel-like factor 4 (KLF4) transcription factor in the terminal differentiation of hematopoietic cells to the monocytes has been well established, the underlying mechanisms remain elusive. To clarify the molecular basis of KLF4-mediated monocytic differentiation, we performed detailed genetic studies in acute myeloid leukemia (AML) cells. Here, we report that dihydropyrimidinase like 2 (DPYSL2), also known as CRMP2, is a novel key differentiation mediator downstream of KLF4 in AML cells. Interestingly, we discovered that KLF4-mediated monocytic differentiation is selectively dependent on one specific isoform, DPYSL2A, but not on other DPYSL family genes. Terminal differentiation to the monocytes and proliferation arrest in AML cells induced by genetic or pharmacological upregulation of KLF4 were significantly reversed by short hairpin RNA (shRNA)-mediated selective depletion of DPYSL2A. Chromatin immunoprecipitation assay revealed that KLF4 associates with the proximal gene promoter of *DPYSL2A* and directly transactivates its expression. Together with the unique expression patterns of *KLF4* and *DPYSL2* limited to the differentiated monocytes in the hematopoietic system both in human and mouse, the identified KLF4-DPYSL2 axis in leukemia cells may serve as a potential therapeutic target for the development of novel differentiation therapies for patients with AML.

## Introduction

Acute myeloid leukemia (AML) is a clonal disorder characterized by differentiation arrest and accumulation of immature myeloid progenitors in the bone marrow, resulting in hematopoietic failure. Despite continuous advances in antitumor strategies, a subset of patients with AML remain refractory to intensive chemotherapy, and others suffer from disease recurrence after achieving remission^1^. Krüppel-like factor 4 (KLF4) is a member of the KLF family of transcription factors that mediate growth inhibition in several types of human cancers, including AML^2–4^. In AML cells, the expression levels of KLF4 are thought to be suppressed by a transcription factor called caudal type homeobox 2^5^. Once activated in AML cells, KLF4 has been shown to function as a potent mediator of terminal differentiation into the monocytes^3,4^. We and other researchers have postulated that upregulation of the transactivation activity of KLF4 in AML cells represent a potential “differentiation therapy” strategy for this disease^3,6^. Although KLF4 has been regarded as a promising therapeutic target in myeloid malignancies, the precise mechanisms of KLF4-mediated tumor suppression or differentiation have been poorly understood.

Dihydropyrimidinase like 2 (DPYSL2), also known as CRMP2, is a member of the collapsin response mediator protein family. Collapsin response mediator proteins form homo- and hetero-tetramers and have been shown to facilitate neuron guidance, growth, and polarity. The encoded protein promotes microtubule assembly and also plays a role in synaptic signaling through interactions with calcium channels. In addition, the expression levels of DPYSL2 have been reported to increase during neural differentiation, and its expression is considered essential in neuronal cell differentiation beyond their signaling function in axon outgrowth^7–10^. This gene has been implicated in multiple neurological disorders, and hyperphosphorylation of its encoded protein may play a key role in the development of Alzheimer's disease^9,11,12^. Alternatively spliced transcript variants encoding multiple isoforms have been observed for this gene, but little is known about their roles in hematopoietic cells or the development and maintenance of leukemia.

Here, we show that KLF4-mediated terminal differentiation of AML cells into the monocytes is mediated by a specific isoform of the *DPYSL2* gene, *DPYSL2A*, thus providing a basis for the development of novel differentiation therapies for AML treatment in the future.

## Results

### KLF4 directly transactivates the expression of DPYSL2A in AML cells

**Figure 1:**
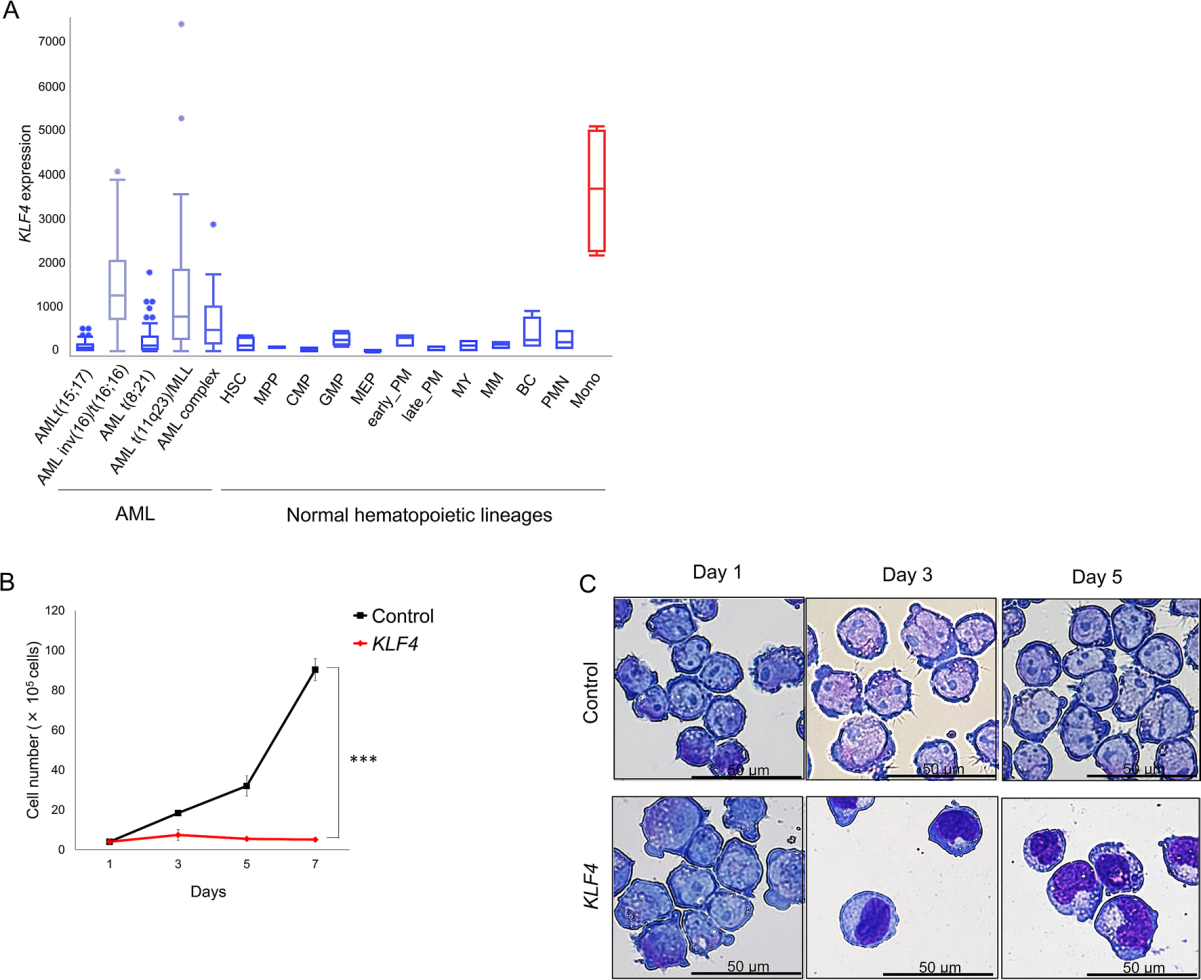
KLF4 induces monocytic differentiation of AML cells. (**A**) Box plot showing the expression levels of KLF4 in primary AML cells (AML t(15; 17), n = 54; AML inv(16)/t(16;16), n = 47; AML t(8;21), n = 60; AML t(11q23)/MLL, n = 43; AML complex, n = 48) or normal hematopoietic cells of various lineages (*HSC* hematopoietic stem and progenitor cells, n = 6; *MPP* multipotent progenitors, n = 2; *CMP* common myeloid progenitors, n = 3; *GMP* granulocyte-monocyte progenitors, n = 7; *MEP* megakaryocyte-erythrocyte progenitors, n = 4; *early_PM* early promyelocytes, n = 3; *late_PM* late promyelocytes, n = 3; *MY* myelocytes, n = 2; *MM* metamyelocytes, n = 3; *BC* band cells, n = 4; *PMN* polymorphonuclear cells, n = 3; *Mono* Monocytes, n = 4) (GSE42519 and GSE13159). Data were retrieved from the BloodSpot database^30^. The database is freely available at www.bloodspot.eu. (**B**) Cell proliferation curves of THP-1 cells transduced with a lentivirus encoding *KLF4* or control cassette. Cells were cultured in the presence of 3 μM doxycycline (n = 3). (**C**) Representative microscopic images of THP-1 cells, as in (**B**). Cells were treated with 3 μM doxycycline for the indicated time periods, harvested, and cytospun onto glass slides. Diff–Quik staining (modified Giemsa staining) was performed on each of the slides (original magnification: ×20, scale bar 50 μm). Data are presented as mean ± SEM. ***P < 0.001, by two-tailed Student’s *t *test.

As we and others have previously reported, increased levels of KLF4 induce terminal differentiation of AML cells into the monocytic lineage^3,4^. We first confirmed that higher levels of KLF4 expression are restricted to the monocytes among all hematopoietic cell lineages in both humans and mice (Fig. 1A, Supplementary Figs. S1B). Consistent with a previous report^4^, the mRNA expression levels of *KLF4* were significantly higher in differentiated monocytes than that in AML cells, suggesting that induction of the expression levels of KLF4 could be a therapeutic strategy for patients with AML (Fig. 1A). Indeed, exogenous expression of KLF4 significantly induced G_1_ phase cell cycle arrest and suppressed the growth of leukemia cells in two different AML cell lines, THP-1 and KO52 (Fig. 1B, Supplementary Figs. S2A, S3). KLF4 expression also led to the terminal differentiation of these AML cells to the monocytes (Fig. 1C, Supplementary Fig. S2B).

**Figure 2:**
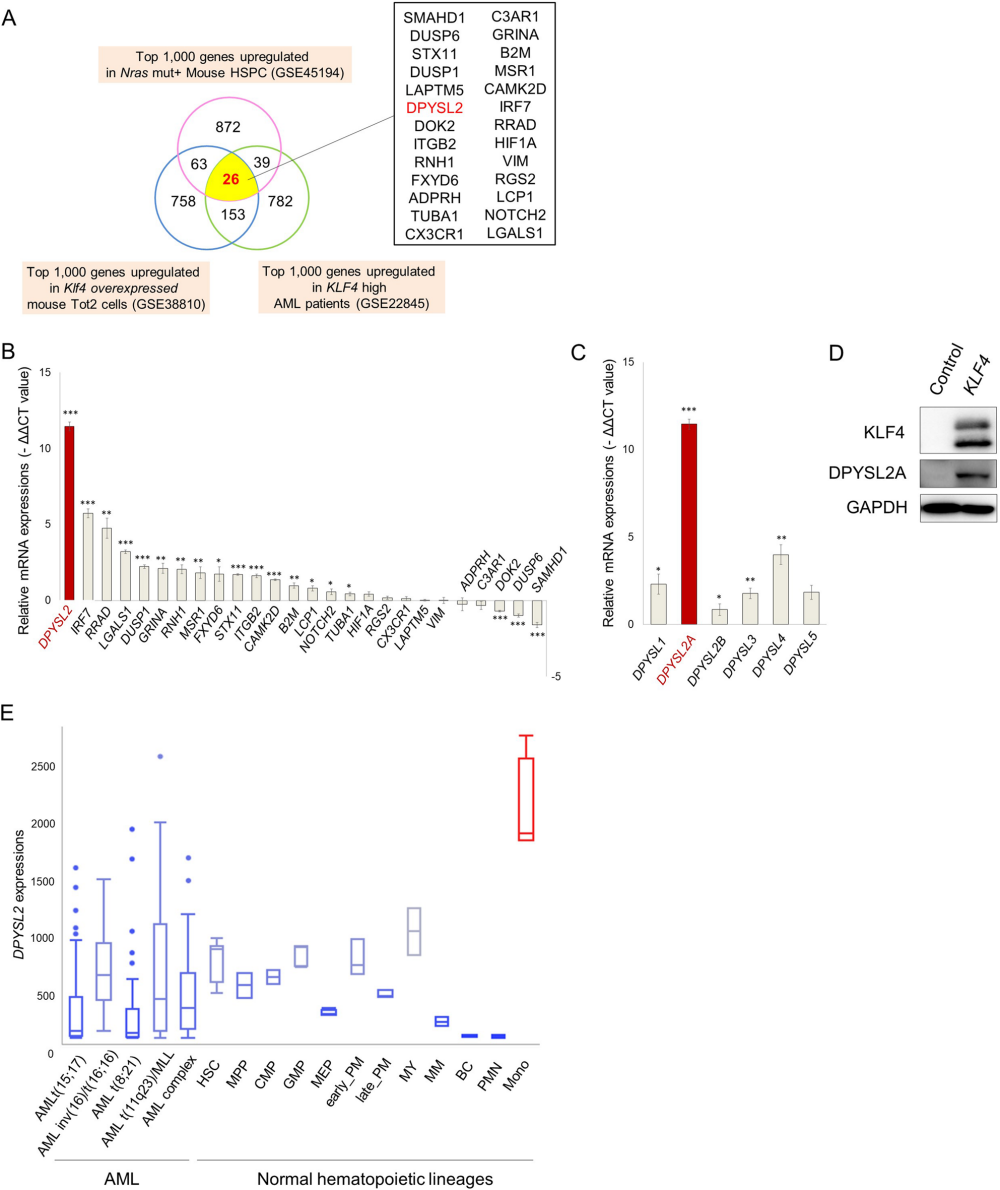
Selective upregulation of DPYSL2A upon exogenous expression of KLF4 in AML cells. (**A**) Identification of genes potentially regulated by KLF4. The top 1000 genes upregulated in *Nras* mut^+^ mouse HSPCs (GSE45194), *Klf4*-overexpressing mouse Tot2 cells (GSE38810), and *KLF4* high-expressing AML patients (GSE22845) were extracted. A list of 26 genes that were commonly upregulated among these three datasets is shown. (**B**) The expression levels of 26 candidate genes from (**A**) in THP-1 cells with exogenous KLF4 expression. Cells were treated with 3 μM doxycycline for 24 h to induce KLF4 expression, and total RNA was extracted and analyzed by qRT-PCR. Values are normalized to those of control vector-transduced cells (n = 3). (**C**) The expression levels of DPYSL family members in THP-1 cells with exogenous KLF4 expression. Cells were treated with 3 μM doxycycline for 24 h to induce KLF4 expression, and total RNA was extracted and analyzed by qRT-PCR. Values are normalized to those of control vector-transduced cells (n = 3). (**D**) Immunoblot analysis of KLF4, DPYSL2A, and GAPDH in THP-1 cells transduced with a lentivirus encoding *KLF4* or control cassette. Cells were treated with 3 μM doxycycline for 48 h and then lysed for protein extraction. (**E**) Box plot showing the expression levels of DPYSL2 in primary AML cells (AML t(15; 17), n = 54; AML inv(16)/t(16;16), n = 47; AML t(8;21), n = 60; AML t(11q23)/MLL, n = 43; AML complex, n = 48) or normal hematopoietic cells of various lineages (*HSC* hematopoietic stem and progenitor cells, n = 6; *MPP* multipotent progenitors, n = 2; *CMP* common myeloid progenitors, n = 3; *GMP* granulocyte-monocyte progenitors, n = 7; *MEP* megakaryocyte-erythrocyte progenitors, n = 4; *early_PM* early promyelocytes, n = 3; *late_PM* late promyelocytes, n = 3; *MY* myelocytes, n = 2; *MM* metamyelocytes, n = 3; BC, band cells, n = 4; *PMN* polymorphonuclear cells, n = 3; *Mono* monocytes, n = 4) (GSE42519 and GSE13159). Data were retrieved from the BloodSpot database^30^. The database is freely available at www.bloodspot.eu. Data are presented as mean ± SEM. *P < 0.05, **P < 0.01, ***P < 0.001, by two-tailed Student’s *t* test.

To identify essential differentiation mediators downstream of *KLF4* in AML cells, we first analyzed the previously reported gene expression microarray data sets. To begin with, we compared the expression levels of each of the genes in mouse myeloid progenitor Tot2 cells with and without exogenous *Klf4* expression (GSE38810) and extracted the top 1000 upregulated genes associated with exogenous *Klf4* expression. Interestingly, continuous stimulation of the mitogen-activated protein kinase (MAPK) signaling cascade has been shown to activate hematopoietic stem and progenitor cells (HSPCs) with increased numbers of differentiated progenitors^13^. Because chronic activation of MAPK signaling has also been shown to induce KLF4 expression^2,3^, we next analyzed the expression levels of each of the genes in mouse HSPCs with somatic *Nras* mutation (GSE45194). We compared the expression levels of each of the genes from wild-type and *Nras* mutation^+^ mice and extracted the top 1000 upregulated genes associated with the *Nras* mutation. In addition, we analyzed the expression levels of each of the genes in human hematopoietic cells from the bone marrow of 154 primary de novo AML patients (GSE22845). We divided the patients into two groups according to their KLF4 expression levels and extracted the top 1,000 upregulated genes that are associated with KLF4 high-expressing AML patients. A Venn diagram was used to identify the overlapping genes in these three data sets, and 26 genes that are the potential downstream targets of KLF4 transcription factor were identified (Fig. 2A; Supplementary Table S1). We then performed qRT-PCR analysis and examined the changes in the expression levels of each of these 26 genes upon exogenous KLF4 expression in THP-1 AML cells. Results showed that 16 out of the 26 genes were significantly upregulated upon forced KLF4 expression. Among the upregulated genes, the expression levels of DPYSL2 were substantially elevated over 2000-fold relative to the baseline (Fig. 2B). Since previous reports have suggested its vital role in neuronal differentiation and polarity as well as axonal growth and guidance, we speculated the possible involvement of DPYSL2 in the regulation of KLF4-mediated monocytic differentiation in AML cells^10,14^. Indeed, the expression levels of DPYSL2 were significantly higher in differentiated monocytes than those in other lineage cells or AML cells (Fig. 2E, Supplementary Fig. S4A,B).

**Figure 3:**
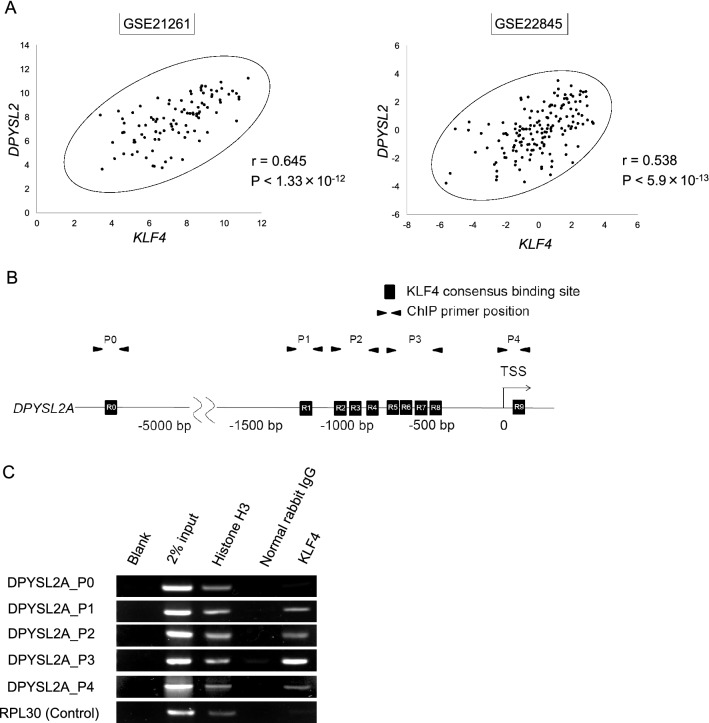
KLF4 directly transactivates DPYSL2A expression in AML cells. (**A**) Correlation between *KLF4* and *DPYSL2* expression in AML patients from 2 independent clinical datasets (GSE22845; n = 154, GSE21261; n = 96). P values were determined by Spearman’s correlation. (**B**) Schematic illustration showing the regulatory region of *DPYSL2A*. There are nine KLF4 consensus binding sites^15,16^ (5′-CACCC-3′, R1-R9) within – 1500 to + 250 bp of the transcription start site (TSS). The positions of the primers used in this study are shown as P0–P4. P0, the region that is 5633 bp upstream of TSS, along with irrelevant RPL30 both serve as controls. The primer sequences are listed in Supplementary Table S4. (**C**) ChIP analysis of THP-1 cells stably transduced with lentivirus encoding *KLF4*. Anti-KLF4 antibody, an isotope-matched control IgG, and anti-Histone H3 antibody were used. Cells were treated with 3 μM doxycycline for 48 h to induce KLF4 expression prior to ChIP assay. ChIP products were subjected to PCR-based amplification with the indicated primer sets, using P0 and RPL30 as negative controls.

The *DPYSL* family of proteins is encoded by seven distinct genes, *DPYSL1*, *DPYSL2A* (*DPYSL2* isoform 1), *DPYSL2B* (*DPYSL2* isoform 2), *DPYSL2C* (*DPYSL2* isoform 3), *DPYSL3*, *DPYSL4*, and *DPYSL5*. DPYSL2B and DPYSL2C differ in the 5′ untranslated region (5′UTR) compared to DPYSL2A and lack a portion of the 5′-coding region, and thus have a shorter N-terminus. Interestingly, qRT-PCR assay in THP-1 and KO52 cells demonstrated that forced expression of KLF4 selectively induced the expression of *DPYSL2A* isoform among the other DPYSL family members (Fig. 2C, Supplementary Fig. S5A). As shown in Fig. 2D and Supplementary Fig. S5B, the protein expression levels of DPYSL2A were also specifically and significantly increased upon exogenous KLF4 expression in THP-1 and KO52 cells, without evident induction of the other isoforms of this gene, DPYSL2B and DPYSL2C. Of note, we identified that the mRNA expression levels of *KLF4* were positively correlated to those of *DPYSL2A*, but not to *DPYSL2B*, in 14 different AML cell lines (Supplementary Fig. S5C). In addition, by analyzing five independently published gene expression data sets (GSE12417, GSE15434, GSE21261, GSE22845 and GSE37642), we found that the mRNA expression levels of *KLF4* and *DPYSL2* are positively correlated in primary AML cells (Fig. 3A, Supplementary Fig. S6). Analysis of the proximal *DPYSL2A* promoter region [− 1500 bp to + 250 bp of transcription start site (TSS)] revealed multiple KLF4 core consensus binding sequences^15,16^ (5′-CACCC-3′) (Fig. 3B, Supplementary Fig. S7). ChIP assay using anti-KLF4 antibody confirmed the direct association between KLF4 and these sites, indicating the direct transactivation of *DPYSL2A* by KLF4 protein (Fig. 3C).

### KLF4-mediated monocytic differentiation is dependent on DPYSL2A expression in AML cells

**Figure 4:**
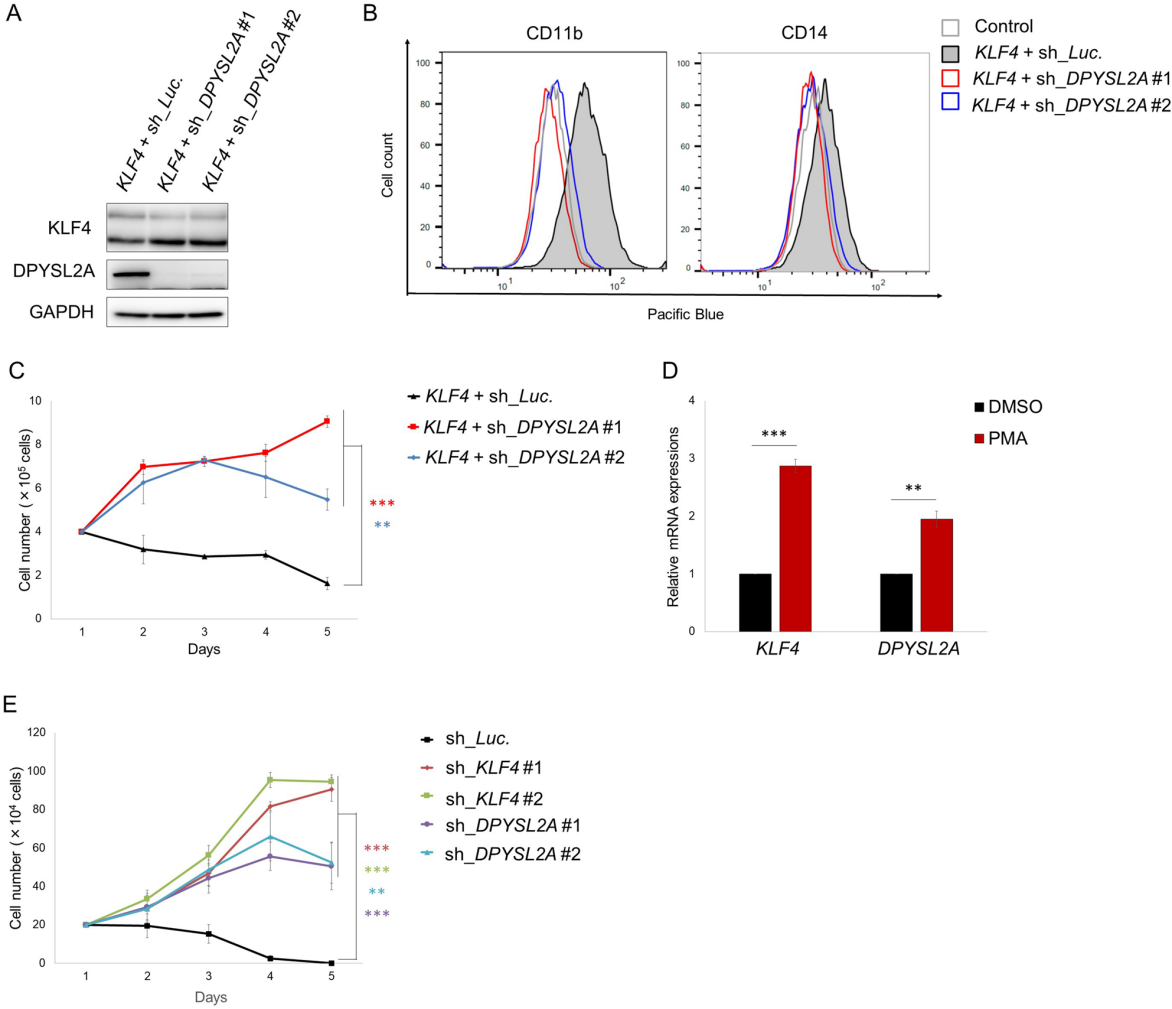
KLF4-mediated monocytic differentiation is dependent on DPYSL2A expression in AML cells. (**A**) Immunoblot analysis of KLF4, DPYSL2A, and GAPDH in THP-1 cells transduced with lentivirus encoding both inducible *KLF4* and shRNAs targeting *DPYSL2A* (sh_*DPYSL2A* #1 and #2) or control luciferase (sh_*Luc*.). Cells were treated with 3 μM doxycycline for 48 h and then lysed for protein extraction. (**B**) Cell surface expression of CD11b and CD14 was determined by flow cytometry in THP-1 cells used in (**A**). Cells were treated with 3 μM doxycycline for 48 h and then harvested for flow cytometric analysis. (**C**) Cell proliferation curves of THP-1 cells used in (**A**). Cells were cultured in the presence of 3 μM doxycycline (n = 3). (**D**) Cumulative expression of KLF4 and DPYSL2A in THP-1 cells treated with PMA (1 ng/mL) for 24 h before RNA extraction. The mRNA expression level of each gene was measured by qRT-PCR (n = 3). (**E**) Growth curves of THP-1 cells transduced with shRNAs targeting *KLF4* (sh_*KLF4* #1 and #2), *DPYSL2A* (sh_*DPYSL2A* #1 and #2), or control luciferase (sh_*Luc*.) in the presence of 0.1 ng/mL PMA. shRNAs were induced by 3 μM doxycycline (n = 3). Data are presented as mean ± SEM. **P < 0.01, ***P < 0.001, by two-tailed Student’s *t* test.

To further demonstrate the importance of DPYSL2A in KLF4-mediated monocytic differentiation in AML cells, we designed doxycycline-inducible shRNAs specifically targeting *DPYSL2A* (sh_*DPYSL2A* #1 and #2) and lentivirally transduced them into THP-1 and KO52 cells. Established THP-1 and KO52 cells were then additionally transduced with a doxycycline-inducible lentiviral DNA construct encoding *KLF4*. Upon doxycycline treatment, these THP-1 and KO52 cells simultaneously expressed KLF4 and shRNAs targeting DPYSL2A (Fig. 4A, Supplementary Fig. S8A). DPYSL2A knockdown reversed KLF4-induced monocytic differentiation in both THP-1 and KO52 cells (Fig. 4B, Supplementary Fig. S8B), as indicated by the increased expression levels of surface markers specific to the differentiated monocytes (CD11b and CD14), and rescued subsequent KLF4-mediated growth inhibition (Fig. 4C, Supplementary Fig. S8C).

Next, we pharmacologically modulated the expression levels of KLF4 within the physiological range using phorbol 12-myristate 13-acetate (PMA) in THP-1 cells. PMA has been shown to induce rapid and sustained activation of Ras-Raf-MEK-ERK pathway, eventually inducing terminal differentiation of AML cells to monocytes through upregulation of KLF4 expression^4^. Consistent with previous reports, PMA treatment rapidly induced KLF4 and DPYSL2A expression in THP-1 cells under our experimental conditions within 24 h (Fig. 4D). Furthermore, continuous exposure to PMA significantly suppressed the growth of THP-1 cells. In contrast, the proliferation of THP-1 cells depleted of KLF4 or DPYSL2A expression was not affected by PMA treatment (Fig. 4E). These results collectively indicate that DPYSL2A is a key mediator of terminal differentiation to monocytes downstream of KLF4.

### DPYSL2A, but not DPYSL2B, mediates monocytic differentiation in AML cells

**Figure 5:**
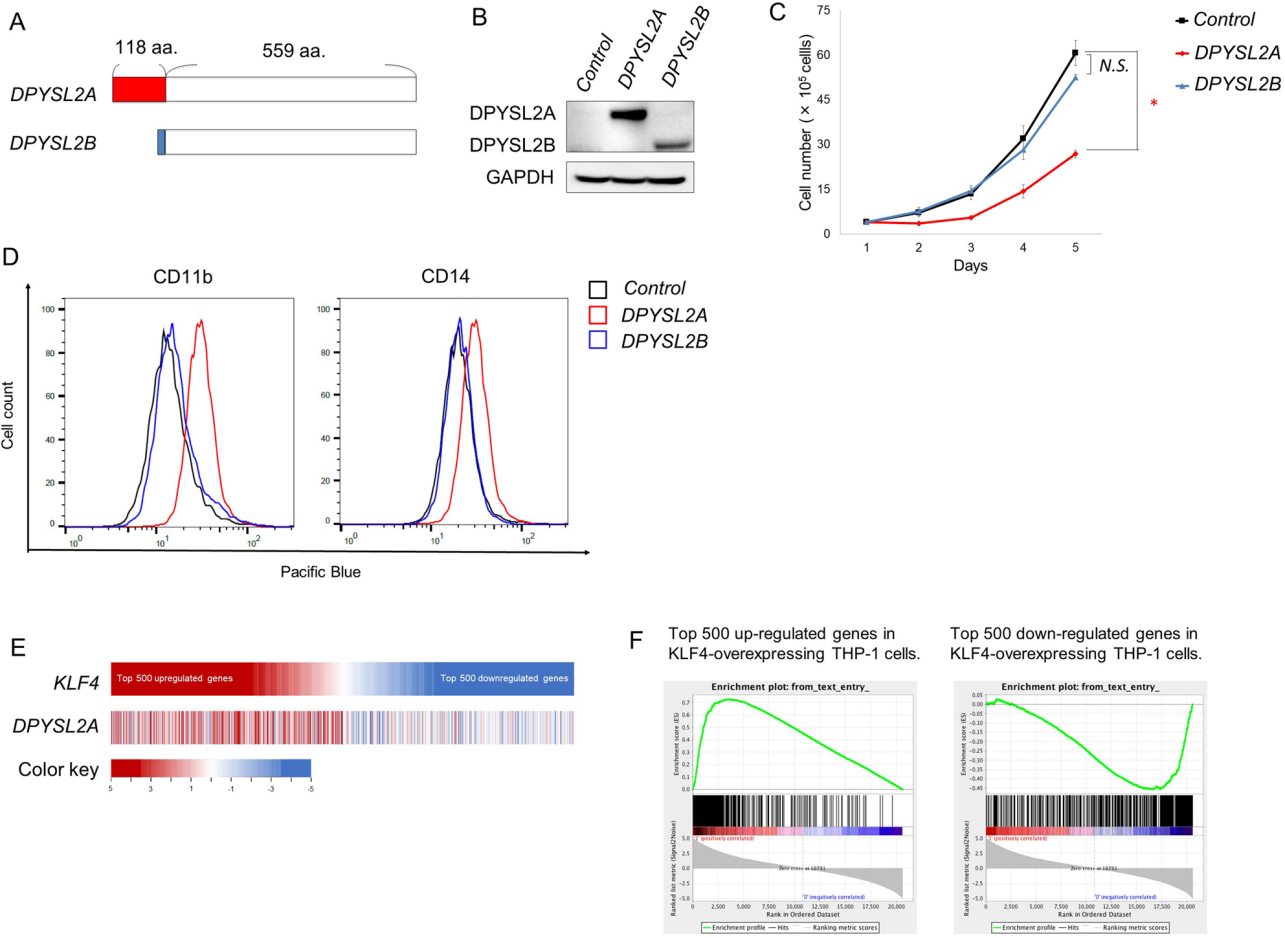
DPYSL2A, but not DPYSL2B induces monocytic differentiation of AML cells. (**A**) Schematic illustration of the DPYSL2A and DPYSL2B genes. DPYSL2A has unique 118 amino acids at its N terminus (shown in red) with common 559 amino acids (shown in white). (**B**) Immunoblot analysis of DPYSL2A, DPYSL2B, and GAPDH in THP-1 cells transduced with lentiviruses encoding *DPYSL2A*, *DPYSL2B*, or control cassette. Cells were treated with 3 μM doxycycline for 48 h and then lysed for protein extraction. (**C**) Cell proliferation curves of THP-1 cells used in (**B**). Cells were cultured in the presence of 3 μM doxycycline (n = 3). (**D**) Cell surface expression of CD11b and CD14 was determined by flow cytometry in THP-1 cells used in (**B**). Cells were treated with 3 μM doxycycline for 48 h and then harvested for flow cytometric analysis. (**E**) Heatmap showing the gene expression profiles of THP-1 cells with exogenous expression of KLF4 or DPYSL2A. The expression levels of the top 500 up- and down-regulated transcripts in KLF4-overexpressing THP-1 cells were examined in DPYSL2A-overexpressing THP-1 cells. Cells were incubated with 3 μM doxycycline for 24 h before total RNA extraction (GSE101751). (**F**) GSEA was performed in THP-1 cells with exogenous DPYSL2A expression to compare the differences in expression of the top 500 transcripts following exogenous expression of KLF4. Each sample was treated with 3 μM doxycycline for 24 h before being lysed for RNA extraction. Data are presented as mean ± SEM. *P < 0.05, two-tailed Student’s *t* test.

To further examine the role of DPYSL2A in AML cells, we prepared THP-1 cells expressing either DPYSL2A or DPYSL2B (Fig. 5A,B, Supplementary Fig. S9). As shown in Fig. 5C,D, exogenous expression of DPYSL2A resulted in monocytic differentiation of THP-1 cells and suppressed cell growth. In contrast, exogenous expression of DPYSL2B, another isoform of DPYSL2 that lacks 118 amino acids in its N terminus, neither induced monocytic differentiation of THP-1 cells nor suppressed cell growth (Fig. 5C,D). A comparison of the gene expression profiles of KLF4- and DPYSL2A-expressing THP-1 cells indicated that the biological roles of these two genes are very closely related in AML cells, at least in the context of terminal differentiation to monocytes (Fig. 5E,F; Supplementary Table S2). Collectively, our study highlighted the importance of the KLF4-DPYSL2A axis in the differentiation of AML cells to the monocytic lineage.

## Discussion

In adults, besides terminally differentiated monocytes^4^, KLF4 is predominantly expressed in the epithelial cells of the gastrointestinal tract^17^, skin^18^, vascular endothelial cells^19^ and thymus^20^. In addition to its documented effect on cell self-renewal and proliferation^21^, KLF4 has been shown to regulate tissue differentiation in these organs^22^. For example, a ~ 90% decrease in the number of differentiated goblet cells was observed in the gastrointestinal tract in *Klf4* knockout mice, without any abnormal changes in the frequency of other cell populations^23^. In addition, in newborn *Klf4*^−/−^ mice, terminal differentiation of the epidermis was disturbed, and mice died shortly after birth due to their inability to maintain a skin barrier and rapid loss of body fluids^18^. These results suggest the crucial role of KLF4 in the differentiation of certain cells, such as colonic epithelial cells and epidermal cells. In this study, we demonstrated the essential role of the transcription factor KLF4 in the terminal differentiation of hematopoietic cells toward the monocytic lineage. The most striking result we present here is that the ability of KLF4 to promote terminal differentiation to the monocytes is highly dependent on the single specific isoform of *DPYSL2*. Since DPYSL2B, not DPYSL2A is the more abundant isoform of DPYSL2 in the tissues studied so far, previous studies have investigated the function of the DPYSL2B isoform^7–10^. Indeed, the biological role of DPYSL2B is well established in neuronal cells, where it regulates axonal growth and differentiation by directly interacting with cytoskeletal proteins, such as tubulin and dynein^7,8^.

DPYSL2A and DPYSL2B transcripts use distinct promoters and differ in their N-terminal amino acid sequences. The DPYSL2B transcript encodes a protein of 572 amino acids, whereas the DPYSL2A transcript encodes a longer protein of 677 amino acids. The two DPYSL2 isoforms have an identical C-terminal 559 amino acids sequence and differ only in their N-terminals. Considering that exogenous expression of only DPYSL2A induces monocytic differentiation in AML cells, the unique N-terminal sequence of 118 amino acids in DPYSL2A might play important roles in mediating this process in hematopoietic cells, but not other cells, such as neuronal cells, where DPYSL2B is dominant and mediates cell differentiation^24^.

From a clinical perspective, our findings provide significant insights for translational researchers to develop novel therapeutic strategies for the treatment of patients with AML. Since recent discoveries and the emergence of new therapeutic agents have bypassed AML cases with poor prognosis, development of alternative therapeutic strategies based on novel drug mechanisms is imperative for this subset of patients. The use of all-trans retinoic acid and arsenic trioxide in acute promyelocytic leukemia has proven that differentiation therapy may significantly improve the survival of patients with AML; however, its success has not been translated to other groups of AML^25^. Therefore, the identification of new therapeutic agents that may induce the differentiation of AML blasts represents an attractive new target, such as the KLF4-DPYSL2 axis identified in this study. Currently, there are no clinically applicable agents available to stimulate this axis. The only experimentally validated KLF4-inducing compound with differentiation potential toward monocytes in AML cells is PMA, which is unsuitable for clinical use due to its serious systemic side effects^26^. This immediate demand for KLF4-DPYSL2A axis-activating compounds should be addressed in future studies, which may help treat otherwise incurable subsets of AML patients.

Taken together, we identified the crucial role of the KLF4-DPYSL2A axis in the terminal differentiation of AML cells to the monocytes in this study. Further studies are required for the better understanding of its precise underlying molecular mechanism. Moreover, developing tools to pharmacologically stimulate the KLF4-DPYSL2A axis in leukemia cells should be enthusiastically explored to establish a novel “differentiation therapy” for patients with AML.

## Methods

### Cell line

AML-derived THP-1 and KG-1a cells were purchased from RIKEN biological resource center (BRC, Japan). AML-derived Kasumi-1, HL60, SKNO-1, NOMO-1 cells and embryonic kidney derived HEK293T cells were from Japanese Collection of Research Bioresources (JCRB, Japan). AML-derived KO52, NB4, ME-1, ML-2, OCI-AML2, MV4-11, MOLM-13 and HEL cells were purchased from Deutsche Sammlung von Mikroorganismen und Zellkulturen GmbH (DSMZ, Germany). HEK293T cells were maintained in Dulbecco's modified Eagle’s medium (DMEM) supplemented with 10% heat-inactivated fetal bovine serum (FBS) and 1% Penicillin–Streptomycin (PS) in a humidified incubator with 5% CO2 and 95% air at 37 °C. The other cells were cultured in Roswell Park Memorial Institute (RPMI) 1640 medium containing 10% FBS and 1% PS under 5% CO_2_ and 95% air at 37 °C.

### Real-time quantitative polymerase chain reaction (qRT-PCR)

Total RNA was isolated using the RNeasy mini kit (Qiagen, USA) and reverse-transcribed with a reverse script kit (TOYOBO, Japan) to generate cDNA. qRT-PCR was performed on a 7500 Real-Time PCR System (Applied Biosystems, USA) according to the manufacturer’s recommendations. The results were normalized to the expression levels of *glyceraldehyde-3-phosphate dehydrogenase* (*GAPDH)*. Relative expression levels were calculated using the 2^–ΔΔCt^ method^27^. The primers used for qRT-PCR are listed in Supplementary Table S3.

### Chromatin immunoprecipitation-quantitative polymerase chain reaction (ChIP-qPCR)

ChIP assay was performed using the SimpleChIP Plus Enzymatic Chromatin IP Kit (Cell Signaling Technology, USA) according to the manufacturer’s protocol. In brief, cells were cross-linked in 1% formaldehyde in phosphate buffered saline for 10 min at room temperature. After glycine quenching, cell pellets were collected, lysed, and then subjected to sonication with the Q55 sonicator system (QSONICA, USA). The supernatant was diluted with the same sonication buffer and processed for immunoprecipitation with an anti-KLF4 antibody (ab106629; Abcam, USA) at 4 °C overnight. The agarose beads were then added, followed by washing with wash buffer as recommended. Chromatin DNA was reverse cross-linked and purified by ethanol precipitation. Following ChIP, the precipitated DNA was quantified by qPCR using the standard procedures for the 7500 Real-Time PCR System (Applied Biosystems, USA). The primers used for ChIP-qPCR are listed in Supplementary Table S4.

### siRNA interference

Specific short hairpin RNAs (shRNAs) targeting human *KLF4* and *DPYSL2A* were designed and sub-cloned into pENTR4-H1tetOx1 and CS-RfA-ETV vectors, respectively. These vectors were kindly provided by Dr. H. Miyoshi (RIKEN BRC, Japan). Non-targeting control shRNA was designed against *luciferase* (sh_*Luc*). The target sequences are listed in Supplementary Table S5.

### Expression plasmids

Human *KLF4, DPYSL2A,* and *DPYSL2B* cDNAs were amplified by PCR and then inserted into the pENTR1A dual selection vector (Thermo Fisher Scientific, USA) and CSIV-TRE-Ubc-KT expression vectors. CSIV-TRE-Ubc-KT was kindly provided by Dr. H. Miyoshi (RIKEN BRC, Japan). All PCR products were verified by DNA sequencing.

### Lentivirus production and transduction

The production of lentivirus was performed as previously described^28^. Briefly, HEK293T cells were transiently co-transfected with lentiviral vectors, psPAX2 and pMD2.G by polyethylenimine (Sigma-Aldrich, USA). Forty-eight hours after transfection, viral supernatants were collected and immediately used for infection. Successfully transduced cells were sorted using the Aria III flow cytometer at the Medical Research Support Center, Graduate School of Medicine, Kyoto University. The Medical Research Support Center was supported by the Platform Project for Supporting Drug Discovery and Life Science Research [Basis for Supporting Innovative Drug Discovery and Life Science Research (BINDS)] from AMED (grant number JP20am0101092).

### Immunoblotting

Immunoblotting was performed as described previously^3^. Membranes were probed with the following primary antibodies: anti-KLF4 (#4038; Cell Signaling Technology, USA), anti-GAPDH (FL-335; Santa Cruz Biotechnology, USA), and anti-DPYSL2 (HPA002381; Sigma-Aldrich, USA). Horseradish peroxidase (HRP)-conjugated anti-rabbit IgG and anti-mouse IgG (Cell Signaling Technology, USA) were used as secondary antibodies. Blots were visualized using Chemi-Lumi One Super (Nacalai Tesque, Japan) and ChemiDoc XRS + Imager (Bio-Rad Laboratories, USA) according to the manufacturer’s recommendations. Protein levels were quantified using the Image Lab Software (Bio-Rad Laboratories, USA).

### Flow cytometry

To assess the monocytic differentiation state of THP-1 and KO52 cells, brilliant violet 421 (BV421)-conjugated monoclonal antibodies specific for human CD11b (ICRF44; BioLegend, USA) and CD14 (HCD14; BioLegend, USA) were used. Flow cytometry analysis was performed using the FlowJo software (BD Biosciences, USA).

### Cell cycle assay

THP-1 cells transduced with the lentivirus encoding *KLF4* were treated with 3 μM doxycycline to induce gene expression. Twenty-four hours after the treatment, cells were fixed in 100% ethanol followed by RNA digestion in PBS containing 100 μg/mL of RNase A. Samples were then stained with 50 μg/mL propidium iodide and immediately subjected to flow cytometric analysis.

### Gene expression microarray analysis

THP-1 cells transduced with the lentivirus encoding *KLF4* or *DPYSL2A* were treated with 3 μM doxycycline for 48 h to induce gene expression. After the indicated incubation time, total RNA was prepared, and its quality was assessed using an Agilent 2100 Bioanalyzer (Agilent Technologies, USA). Using 250 ng of total RNA, biotinylated cRNA was prepared according to the standard Affymetrix protocol with the GeneChip 3′ IVT PLUS Reagent Kit. Following fragmentation, 15 µg of cRNA was hybridized for 16 h at 45 °C on the GeneChip Hybridization Oven 645. GeneChips were washed and stained in the GeneChip Fluidics Station 450 using GeneChip Hybridization, Wash, and Stain Kit. GeneChips were scanned using the GeneChip Scanner 3000 7G. The data were analyzed with Microarray Suite version 5.0 (MAS 5.0) using Affymetrix default analysis settings and global scaling as a normalization method. The trimmed mean target intensity of each array was arbitrarily set to 100. Our microarray data have been deposited in NCBI’s Gene Expression Omnibus (GEO Series accession number GSE101751). Gene set enrichment analysis (GSEA) was utilized to analyze the microarray data obtained in the present study^29^.

### Statistical analysis

Differences between control and experimental groups were assessed by a two-tailed unpaired Student’s *t* test and declared statistically significant if the *p* value was less than 0.05. The equality of variances in two populations was calculated using the F test. The results are represented as the mean ± standard error of mean (SEM) of values obtained from three independent experiments. In transplantation experiments, animals were randomly allocated to the experimental groups and the treatments were given by blinding. Survival rates between the indicated groups were compared using the log-rank test.

### Study approval

We did not perform any experiments involving humans or animals in this study.

## Supplementary information

Supplementary Information.
