# Supplementary material for: Pivotal role of DPYSL2A in KLF4-mediated monocytic differentiation of acute myeloid leukemia cells
Source: Sci Rep. 2020 Nov 20;10:20245. doi: 10.1038/s41598-020-76951-0 (PMC7680118; doi:10.1038/s41598-020-76951-0)
Supplement: Supplementary file 1 — Supplementary Information. [file 41598_2020_76951_MOESM1_ESM.docx]

**Supplementary Information**

**Pivotal Role of DPYSL2A in KLF4-mediated Monocytic Differentiation of Acute Myeloid Leukemia Cells.**

Mina Noura^*^, Ken Morita^*^, Hiroki Kiyose, Hidemasa Matsuo, Yoko Nishinaka-Arai, Mineo Kurokawa, Yasuhiko Kamikubo and Souichi Adachi.

* These authors contributed equally to this work.

Supplementary Figures p. 2

Supplementary Tables p. 13

Uncropped Data p. 41

**Supplementary Figures**

**
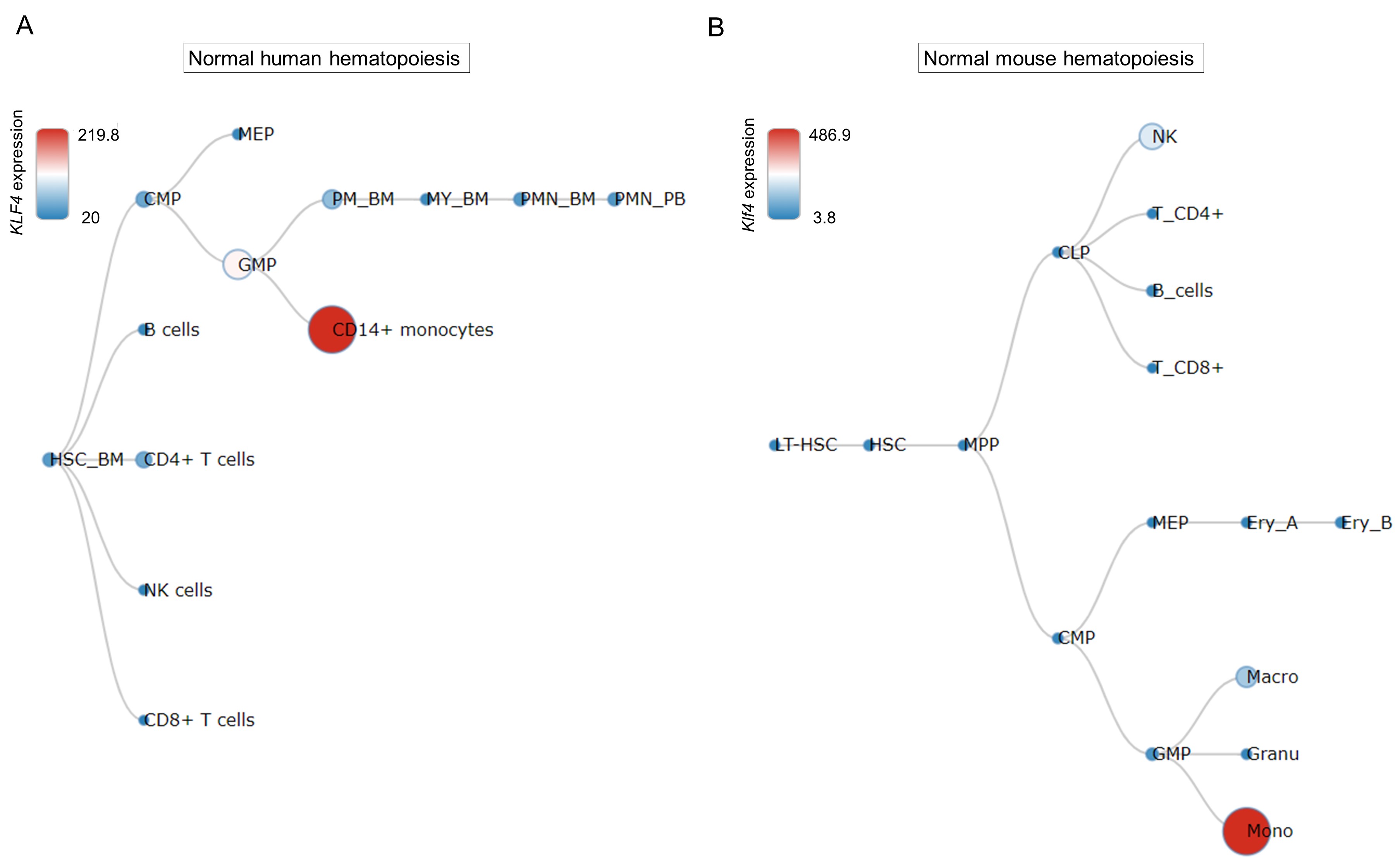
**

**Supplementary Figure 1**

(A) Hierarchical differentiation tree of human hematopoietic cells (HSC_BM, hematopoietic stem cells from bone marrow, n = 8; CMP, common myeloid progenitor cell, n = 3; GMP, granulocyte monocyte progenitors, n = 3; MEP, megakaryocyte-erythroid progenitor cell, n = 3; PM_BM, promyelocyte from bone marrow, n = 3; MY_BM, myelocyte from bone marrow, n = 3; PMN_BM, polymorphonuclear cells from bone marrow, n = 4; PMN_PB, polymorphonuclear cells from peripheral blood, n = 2; CD14+ Monocytes, n = 14; B cells, n = 5; CD4+ T cells, n = 5; CD8+ T cells, n = 5; NK cells, n = 5) showing expression levels of *KLF4* at different maturation stages (GSE17043, GSE19599, GSE11864 and E-MEXP-1242). Data were retrieved from the BloodSpot database^1^. The database is freely available at www.bloodspot.eu

(B) Hierarchical differentiation tree of mouse hematopoietic cells (LT-HSC, long term hematopoietic stem cells, n = 1; HSC, hematopoietic stem cells, n = 4; MPP, multipotent progenitor, n = 2; CLP, common lymphoid progenitor, n = 4; CMP, common myeloid progenitor, n = 4; GMP, granulocyte monocyte progenitors, n = 4; Macro, macrophages, n=4; Granu, granulocytes, n=6; Mono, monocytes, n = 4; B cells, n=2; T_CD4+, n = 4; T_ CD8+, n = 4; NK, n = 2; MEP, megakaryocyte-erythroid progenitor cell, n = 4; Ery_A, Erythrocytes A, n = 2; Ery_B, Erythrocytes B, n = 1) showing expression levels of *Klf4* at different maturation stages (GSE60101). Data were retrieved from the BloodSpot database^1^. The database is freely available at www.bloodspot.eu

**Supplementary Figure 2**

(A) Cell proliferation curves of KO52 cells transduced with a lentivirus encoding KLF4 or control cassette. Cells were cultured in the presence of 3 μM doxycycline (n = 3).

(B) Representative microscopic images of KO52 cells, as in (A). Cells were treated with 3 μM doxycycline for the indicated time periods, harvested, and cytospun onto glass slides. Diff-Quik staining (modified Giemsa staining) was performed on each of the slides (original magnification: 20×, Scale bar: 50 μm).

Data are presented as mean ± SEM. **P < 0.01, by two-tailed Student’s *t-*test.

**Supplementary Figure 3**

KLF4 overexpression induces G1 phase cell cycle arrest. THP-1 cells transduced with lentivirus encoding *KLF4* or control cassette were cultured in the presence of 3 μM doxycycline to induce gene expression. Twenty-four hours after the treatment, cells were harvested and analyzed by flow cytometry (*n* = 3).

Data are presented as mean ± SEM. * P < 0.05, **P < 0.01, *** P < 0.001, by two-tailed Student’s *t-*test.


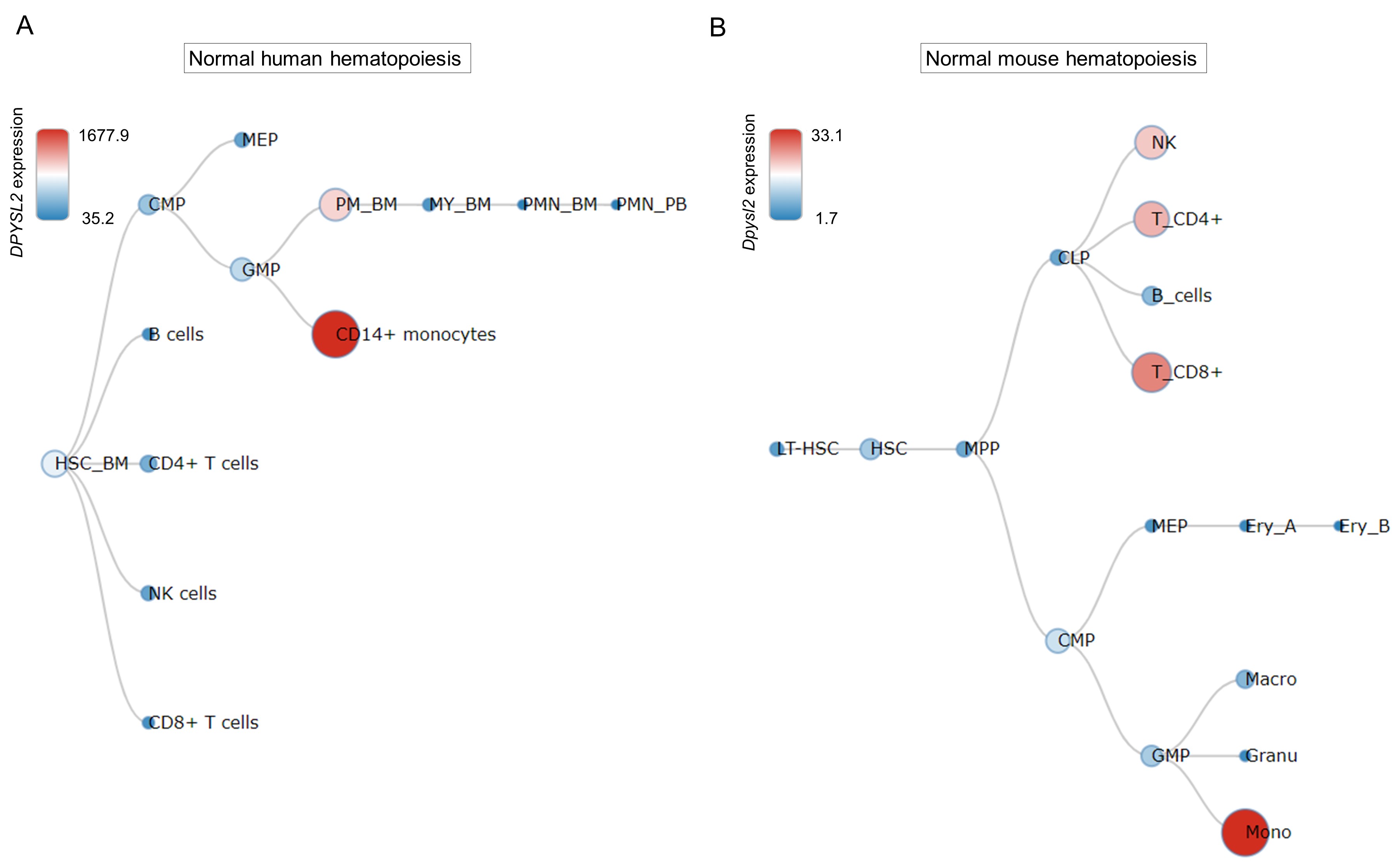


**Supplementary Figure 4**

(A) Hierarchical differentiation tree of human hematopoietic cells (HSC_BM, hematopoietic stem cells from bone marrow, n = 8; CMP, common myeloid progenitor cell, n = 3; GMP, granulocyte monocyte progenitors, n = 3; MEP, megakaryocyte-erythroid progenitor cell, n = 3; PM_BM, promyelocyte from bone marrow, n = 3; MY_BM, myelocyte from bone marrow, n = 3; PMN_BM, polymorphonuclear cells from bone marrow, n = 4; PMN_PB, polymorphonuclear cells from peripheral blood, n = 2; CD14+ Monocytes, n = 14; B cells, n = 5; CD4+ T cells, n = 5; CD8+ T cells, n = 5; NK cells, n = 5) showing expression levels of *DPYSL2* at different maturation stages (GSE17043, GSE19599, GSE11864 and E-MEXP-1242). Data were retrieved from the BloodSpot database^1^. The database is freely available at www.bloodspot.eu

(B) Hierarchical differentiation tree of mouse hematopoietic cells (LT-HSC, long term hematopoietic stem cells, n = 1; HSC, hematopoietic stem cells, n = 4; MPP, multipotent progenitor, n = 2; CLP, common lymphoid progenitor, n = 4; CMP, common myeloid progenitor, n = 4; GMP, granulocyte monocyte progenitors, n = 4; Macro, macrophages, n=4; Granu, granulocytes, n=6; Mono, monocytes, n = 4; B cells, n=2; T_CD4+, n = 4; T_ CD8+, n = 4; NK, n = 2; MEP, megakaryocyte-erythroid progenitor cell, n = 4; Ery_A, Erythrocytes A, n = 2; Ery_B, Erythrocytes B, n = 1) showing expression levels of *Dpysl2* at different maturation stages (GSE60101). Data were retrieved from the BloodSpot database^1^. The database is freely available at www.bloodspot.eu


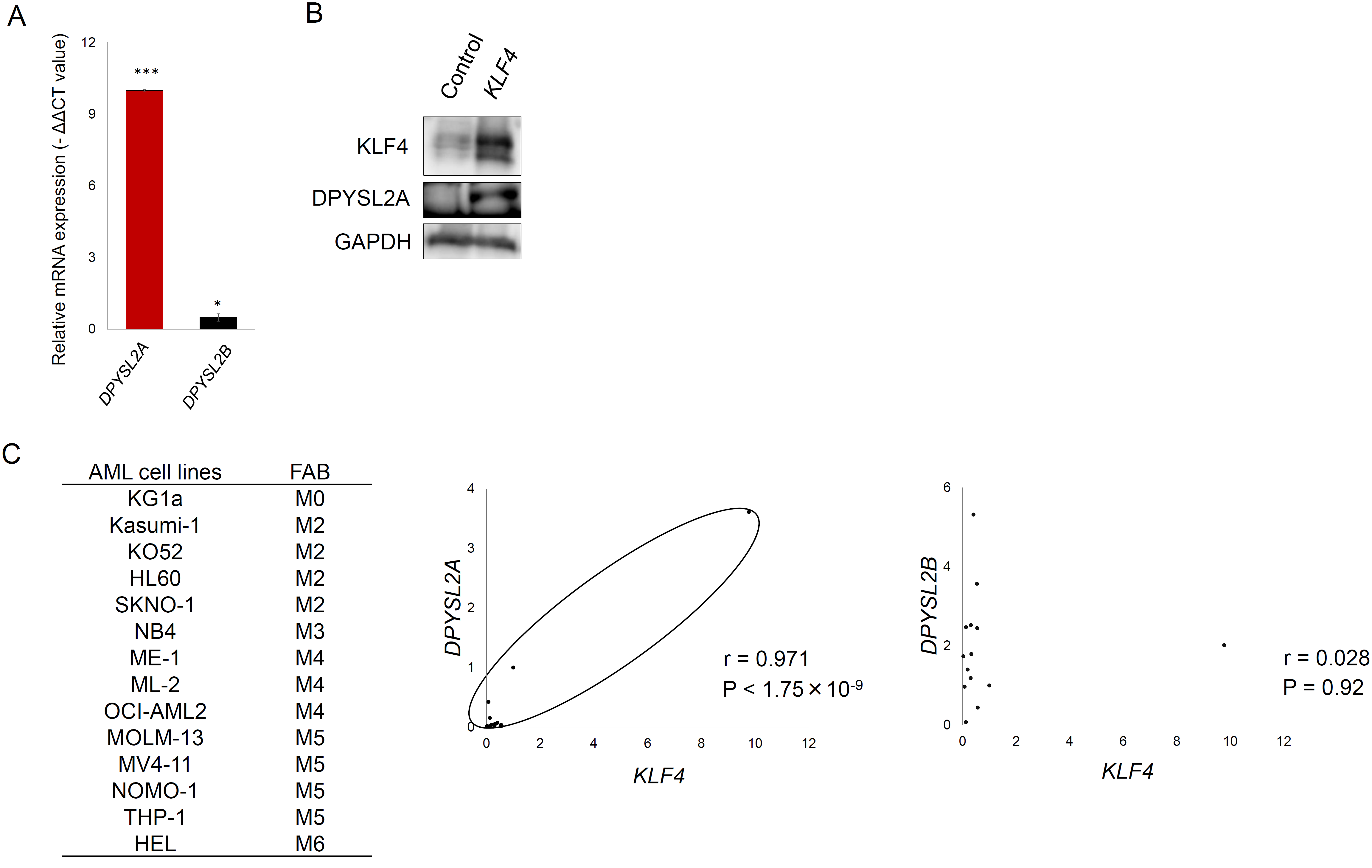


**Supplementary Figure 5**

(A) Expression levels of *DPYSL*2A and *DPYSL*2B in KO52 AML cells with exogenous KLF4 expression. Cells were either transduced with lentivirus encoding *KLF4* or control cassette and treated with 3 μM doxycycline for 24 h, then total RNA was prepared and analyzed by real-time RT-PCR. Values are normalized to that of the control cells (n = 3). Data are presented as mean ± SEM. * P < 0.05, *** P < 0.001, by two-tailed Student’s *t*-test.

(B) Immunoblot of KLF4, DPYSL2A and GAPDH in KO52 cells transduced with lentivirus encoding *KLF4* or control cassette. Cells were treated with 3 μM doxycycline for 48 h, then lysed for protein extraction.

(C) Correlation between the mRNA expression levels of *KLF4* and *DPYSL2A*, or *KLF4* and *DPYSL2B* in various AML cell lines (n = 14). P-values were determined by Spearman’s correlation.

**Supplementary Figure 6**

Correlation between the mRNA expression levels of *KLF4* and *DPYSL2* in three independent datasets of AML patients (GSE12417; n = 79, GSE15434; n = 251, GSE37642; n = 140). P-values were determined by Spearman’s correlation.

**Supplementary Figure 7**

The amino acid sequence of the *DPYSL2A* promoter used in this study [–1,500 bp to +250 bp of transcription start site (TSS)]. The KLF4 core consensus binding sequences (5’-CACCC-3’) are highlighted in red. Each of the primer positions and the amplified regions are highlighted in yellow (P1), green (P2), blue (P3), and gray (P4).


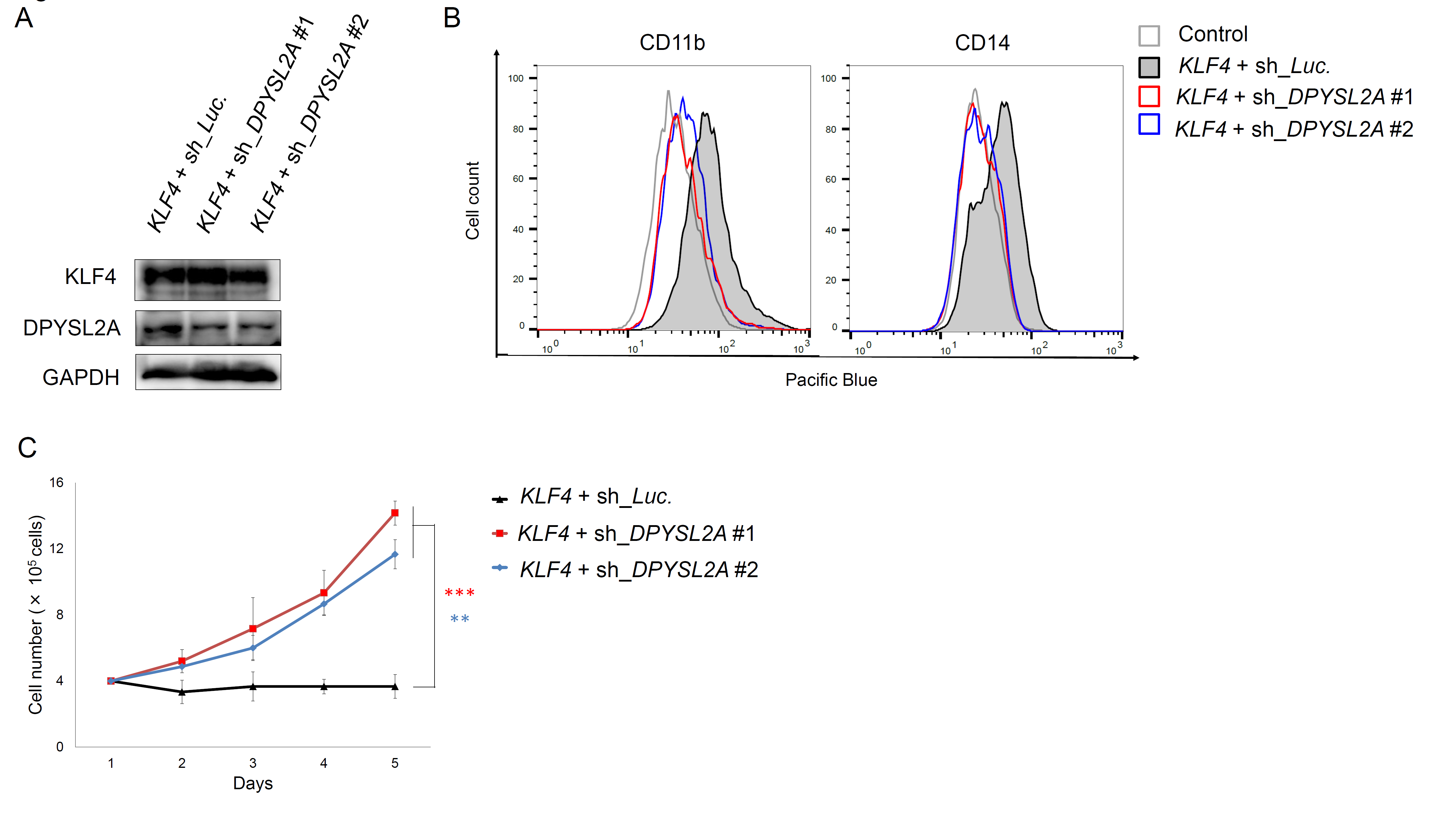


**Supplementary Figure 8**

(A) Immunoblot analysis of KLF4, DPYSL2A, and GAPDH in KO52 cells transduced with lentivirus encoding both inducible *KLF4* and shRNAs targeting *DPYSL2A* (sh_*DPYSL2A* #1 and #2) or control luciferase (sh_*Luc*.). Cells were treated with 3 μM doxycycline for 48 h and then lysed for protein extraction.

(B) Cell surface expression levels of CD11b and CD14 were determined by flow cytometry in KO52 cells used in (A). Cells were treated with 3 μM doxycycline for 48 h and then harvested for flow cytometric analysis.

(C) Cell proliferation curves of KO52 cells used in (A). Cells were cultured in the presence of 3 μM doxycycline (n = 3).

Data are presented as mean ± SEM. **P < 0.01, ***P < 0.001, by two-tailed Student’s *t*-test.


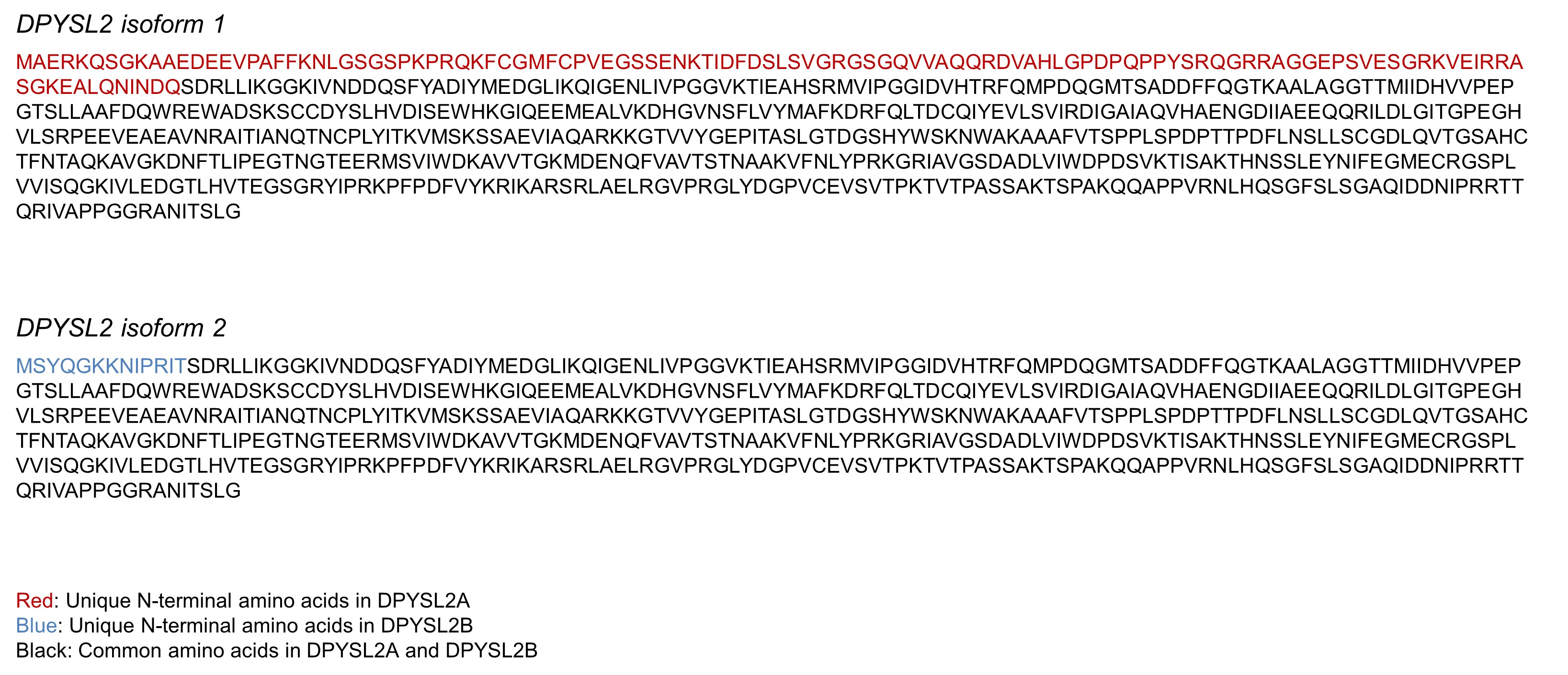


**Supplementary Figure 9**

Amino acid sequences of DPYSL2 isoform 1 (DPYSL2A) and DPYSL2 isoform 2

(DPYSL2B). DPYSL2A has distinctive 118 amino acids N-terminus sequences (shown

in red) compared to DPYSL2B.

**References**

1 Bagger, F. O., Kinalis, S. & Rapin, N. BloodSpot: a database of healthy and malignant haematopoiesis updated with purified and single cell mRNA sequencing profiles. *Nucleic Acids Res* **47**, D881-D885, doi:10.1093/nar/gky1076 (2019).

**Supplementary Tables**

**Supplementary Table 1**

Candidate gene list related to Fig. 2A.

**Supplementary Table 2**

List of top 500 genes regulated in KLF4-overexpressed THP-1 cells related to Figs 5E and 5F.

**Supplementary Table 3**

PCR primers used for RT-qPCR experiments.

**Supplementary Table 4**

PCR primers used for ChIP experiments.

**Supplementary Table 5**

Target sequences for shRNA knockdown experiments.

**Uncropped Data**

**Figure 2D**

**Figure 3C**

-

**Figure 4A**

**Figure 5B**

**Figure S5B**

**Figure S8**
